# Non-invasive assessment of vibration perception and protective sensation in people with diabetes mellitus: inter- and intra-rater reliability

**DOI:** 10.1186/s13047-020-0371-9

**Published:** 2020-01-16

**Authors:** Sean Michael Lanting, Martin Jeremy Spink, Peta Ellen Tehan, Stephanie Vickers, Sarah Louise Casey, Vivienne Helaine Chuter

**Affiliations:** 1 School of Health Sciences, University of Newcastle, Newcastle, Australia; 2 Priority Research Centre for Gender, Health and Ageing, University of Newcastle, Newcastle, Australia; 3 Priority Research Centre for Physical Activity and Nutrition, University of Newcastle, Newcastle, Australia

**Keywords:** Diabetes, Neuropathy, Reliability, Monofilament, Vibration, Tuning fork, Neurothesiometer

## Abstract

**Background:**

Testing of protective sensation and vibration perception are two of the most commonly used non-invasive methods of screening for diabetes-related peripheral neuropathy (DPN). However, there is limited research investigating the reliability of these tests in people with diabetes. The aim of this study was to determine the inter- and intra-rater reliability of methods used to test vibration perception and protective sensation in a community-based population of adults with type 2 diabetes.

**Methods:**

Three podiatrists with varying clinical experience tested four- and 10-site, 10 g monofilament and vibration perception threshold (VPT). In a separate cohort, the reliability of a graduated tuning fork as well as two methods of conventional tuning fork (on/off method and dampening method) was undertaken by a new graduate podiatrist and podiatrist with one-year’s clinical experience. The intra- (Cohen’s К) and inter-rater (Cohen’s or Fleiss’ К) reliability of each test was determined.

**Results:**

Fifty participants (66% male, 100% type 2, 32% with DPN) underwent monofilament and neurothesiometer testing with 44 returning for the retest. Twenty-four participants (63% male, 100% type 2, 4% with DPN) underwent tuning fork testing and returned for retest. All tests demonstrated acceptable inter-rater reliability ranging from moderate (10-site monofilament, К: 0.54, CI: 0.38–0.70, *p* = 0.02) to substantial (graduated tuning fork, К: 0.68, CI: 0.41–0.95, *p* < 0.01). The 10-site monofilament (К: 0.44–0.77) outperformed the 4-site test (К: 0.34–0.67) and the dampened tuning fork method (К: 0.41–0.49) showed lower intra-rater reliability compared to both conventional (К: 0.52–0.57) and graduated methods (К: 0.50–0.57).

**Conclusion:**

We support the current recommendations of using more than one test to screen and monitor progression of DPN. Four- and 10-site 10 g monofilament testing have similarly acceptable levels of reliability and the neurothesiometer is the most reliable method of assessing vibration perception function. Use of a graduated tuning fork was slightly more reliable than other methods of tuning fork application however all had substantial reliability. Years of clinical experience only marginally affected test reliability overall and due to subjective nature of the tests we suggest that testing should be performed regularly and repetitively.

## Background

Diabetes is a significant health problem and was recently estimated to affect approximately 451 million people worldwide [1]. Up to 50% of persons with diabetes are affected by diabetic peripheral neuropathy (DPN), which causes widespread sensory loss, primarily affecting the feet and legs [2–5]. DPN is associated with lower limb complications such as foot deformity [6], increased plantar pressures [7], ulceration and infection and, is implicated in 50–75% of all non-traumatic lower limb amputations [8]. Prophylactic care in people with diabetes has been shown to prevent or delay development of DPN. For example, intensive glycaemic control has demonstrated a reduction of neuropathy incidence of between 25% [9] and 57% [10]. Additionally education and routine foot care in those with DPN have been shown to reduce risk of associated foot complications [11, 12]. Therefore, early and accurate diagnosis of DPN is paramount to mitigating the risk of associated foot complications.

Methods for conducting clinical chairside neurological tests to establish the presence and monitor the progression of DPN are varied, and assess different nerve fibre types. Current international guidelines recommend testing of protective sensation using monofilament, as well as additional tests such as vibration perception, reflexes, pain perception and asking about neurological symptoms [13, 14]. Diminished vibration perception and ability to detect 10 g monofilament have demonstrated predictive capacity for future foot ulceration [8, 15–18], and are widely used both clinically and in research. Several techniques are available for testing vibration perception, including use of a neurothesiometer or similar instrument, as well as graduated and non-graduated tuning forks. Similarly, methods for testing protective sensation testing using monofilament examination can vary clinically in terms of location and number of sites tested. However there are limited data available comparing the reliability of different testing methods. Reliability refers to the level of consistency of measurement results between different clinicians (inter-rater) and the same clinican on multiple occasions (intra-rater). While there have been several small studies investigating inter- and/or intra-rater reliability of monofilament [19–21] and vibration perception testing [21–25] results of these studies are variable, and generalisability of these findings limited by inconsistency of testing methods. One larger study recently compared effectiveness of three, 4 and 10 site monofilament for identifying DPN in 1915 people with diabetes, and in doing so, reported high level of agreement between testing methods (К: 0.797 to 0.925) [26], but did not report reliability on individual tests.

The aim of this study was to determine the inter- and intra-rater reliability of commonly used testing methods of protective sensation and vibration perception, performed by podiatrists with varying amounts of clinical experience, in people with diabetes. Specifically, a four-site and a 10-site monofilament test, as well as vibration perception as determined by neurothesiometer, graduated tuning fork and non-graduated (dampened and conventional methods) tuning fork.

## Methods

This study was conducted at the University of Newcastle Podiatry clinics in New South Wales, Australia. Ethics approval was obtained from the University of Newcastle Human Research Ethics Committee prior to undertaking this study, protocol code H-2012-0141. All participants involved in this study provided written informed consent prior to study commencement.

### Participants

Participants were recruited on a volunteer basis, with flyers posted up in university clinic consultation rooms and the waiting room, directing potential recruits to register their interest. Recruitment was performed by people who were not involved in test performance thereby ensuring blinding of raters to participant health status. Participants included in the study were required to be representative of the population in which screening for DPN is recommended [14]. Therefore, inclusion criteria were Type-1 diabetes of five years or more or Type-2 diabetes of any duration with and without history of diagnosed DPN, confirmed by medical records. Participants were required to be fluent in English language to satisfy consent for the study. Exclusion criteria included active foot ulceration, visual evidence of recently healed foot ulceration, lower limb amputation of any kind or diagnosed peripheral neuropathy of an origin other than diabetes.

The inter- and intra-rater reliability of 10 g monofilament testing using four-site and 10-site testing techniques as well as vibration perception threshold (VPT) using a neurothesiometer were determined across three raters [a new graduate podiatrist (R1); a podiatrist with five years of clinical experience (R2); and a podiatrist with 10 years of clinical experience (R3)]. In addition, inter- and intra-rater reliability of a graduated tuning fork as well as an on/off and a dampened method of a conventional tuning fork were tested in a podiatrist with one year’s clinical experience (R4) and a new graduate podiatrist (R5).

## Testing methods

### Monofilament testing

Semmes-Weinstein 10 g monofilaments (North Coast Medical, California) were used to conduct all monofilament testing. A four-site [27] and a ten-site monofilament [8] test were used. For the four-site test, site application was plantar surface of the hallux as well as first, third and fifth metatarsal heads, while the 10-site test also included the plantar surface of the third and fifth digits, heel, medial arch, lateral arch and the dorsal surface of the mid foot. Perception of six or less sites in the 10-site test [28] and three or less sites in the four-site test [8] were considered abnormal. Monofilaments were applied perpendicular to the skin until buckling and held in place for 1–2 s. The participants were asked to respond with a “yes” on each occasion where they could perceive the 10 g force. Monofilaments used in this study were discarded following use on nine consecutive participants ensuring they were not used more than 100 times within 24 h in order to maintain the force applied at 10 g [29].

### Neurosthesiometer testing

Horwell neurothesiometers (Wilford Industrial, Nottingham) were used to determine vibration perception threshold (VPT)*.* The stylus of the device was applied to the apex of the right hallux and the amplitude of vibration of the device was then gradually increased until the participant could perceive the vibration. The corresponding VPT value was immediately written on the assessment form and the process repeated until three values were recorded. The mean of the three values was calculated, with a mean VPT value > 25 v considered an abnormal response [27] .

### Tuning fork testing

The vibration perception tests were performed using graduated C64-Hz Ragg Rydel-Seiffer (Granton Medical, Sheffield) and conventional (non-graduated) Ragg Gardiner Brown C128-Hz (Granton Medical, Sheffield) tuning forks. For each tuning fork test a manually applied force to induce vibration was applied to the tines of the tuning fork before placing the device on the apex of the right hallux. Participants were instructed to indicate verbally when they felt vibration and then when they perceived the vibration had stopped (C128-Hz). If the participant could not detect the vibration at all then it was considered abnormal (on/off method) [17]. Once the vibration was perceived, the rater would randomly dampen the tuning fork (C128-Hz) with their other hand and if the participant could not perceive that the vibration had stopped then this was considered an abnormal response (dampening method) [23]. Lastly, perception of less than four octals as quantified by the graduated tuning fork (C64 Hz) constituted an abnormal response [30].

## Testing protocol

In both the initial testing session and retest for all testing conducted as part of this study, raters performed the relevant neurological tests in a pre-determined random order on every participant in separate treatment rooms. Raters were blinded to the participant health status i.e. presence, absence, or extent of DPN, though were aware that all of the participants had diabetes. Raters were also blinded to each other’s results as well as to their own results from the first testing session when undertaking the retest. The order of application of the tests was randomised using an online random number generator (www.randomizer.org). The order of raters was randomised in a manner that was not pre-determined and the order of site application of the monofilament was randomised at the discretion of the individual raters. Participants were blind to all results, though were provided with a plain language summary on request at study completion. The tests were performed only on the right limb in order to satisfy the assumption of independence of data [31], with the right limb chosen rather than a random limb in order to minimise rater confusion. Participants were required to attend the retest after seven days at the same location and were required to close their eyes for each test procedure. In addition, each test was first demonstrated on the dorsal aspect of the participant’s hand and in relation to vibration, ‘buzzing’ was differentiated from pressure sensation.

### Statistical analysis

SPSS version 25 was used for statistical analysis. Results for all neurological tests were broken down into dichotomous variables, namely abnormal or normal results, with abnormal being indicative of neuropathy. The intra-rater reliability was calculated using an unweighted Cohen’s Kappa (К) statistic [32]. In order to calculate the inter-rater reliability and effect of experience on reliability, Cohen’s К was initially determined between the following pairs of raters: R1 and R2; R1 and R3; and R2 and R3 (monofilament and neurothesiometer) and R4 and R5 (tuning fork tests). Fleiss’ К was then calculated to determine the overall reliability between raters R1-R3 [33]. Interpretation of the Cohen’s and Fleiss’ К statistic was performed using the method proposed by Landis and Koch [34] (Values indicating: 0.01–0.20 = slight, 0.21–0.40 = fair, 0.41–0.60 = moderate, 0.61 to 0.80 = substantial, and 0.81–1.0 = almost perfect). Values below 0.4 were interpreted as clinically unacceptable for reliability of a test [35].

## Results

Fifty participants volunteered for testing with monofilament and neurothesiometer, of whom 44 returned for the retest. Six participants were unable to return within the required period of seven days and thus did not take part in the intra-rater reliability component of this study. Twenty-four participants volunteered for tuning fork testing, all of whom returned for the re-test. Participant characteristics are detailed in Table 1.

**Table 1:** Participant characteristics

Monofilament and VPT (*n* = 50)				
	N	Mean Age (SD)	T2-DM	Existing DPN diagnosis
Males	33	72 (11)	33	16
Females	17	73 (7)	17	0
Tuning fork (*n* = 24)				
	N	Mean Age (SD)	T2-DM	Existing DPN diagnosis
Males	15	74 (7)	15	1
Females	9	70 (9)	9	0

*SD* standard deviation*, T2* DM type two diabetes*, DPN* diabetic peripheral neuropathy

### Monofilament

Intra-rater reliability: The four-site 10 g monofilament examination demonstrated variable intra-rater reliability (*n* = 50) with Cohen’s К ranging from fair (К = 0.34, 95%CI: 0.06 to 0.63, *p* = 0.02) to substantial (К = 0.67, 95%CI: 0.45 to 0.89, *p* < 0.01), Table 2. The 10-site monofilament test demonstrated intra-rater reliability (n = 50) ranging from moderate (К = 0.44, 95%CI: 0.09 to 0.79, *p* < 0.01) to substantial (К = 0.77, p5%CI: 0.55 to 0.99, *p* < 0.01) and was not related to increasing clinical experience, Table 2.

**Table 2:** Intra-rater reliability reported as Cohen’s К and SE with 95%CI and *p*-values

Monofilament and VPT (*n* = 44)												
	Rater 1				Rater 2				Rater 3			
Test	K	SE	95% CI	P	K	SE	95% CI	P	K	SE	95% CI	P
VPT	0.52	0.15	0.21 to 0.82	0.01	0.78	0.10	0.58 to 0.98	0.02	0.72	0.13	0.46 to 0.98	< 0.01
Monofilament [4]	0.44	0.14	0.16 to 0.73	0.02	0.67	0.11	0.45 to 0.89	< 0.01	0.34	0.15	0.06 to 0.63	0.02
Monofilament [10]	0.49	0.18	0.14 to 0.83	< 0.01	0.77	0.11	0.55 to 0.99	< 0.01	0.44	0.18	0.09 to 0.79	< 0.01
Tuning fork (*n* = 24)												
	Rater 4				Rater 5							
Test	K	SE	95% CI	P	K	SE	95% CI	P				
Graduated	0.57	0.17	0.24 to 0.9	< 0.01	0.50	0.17	0.17 to 0.83	< 0.02				
Conventional (on/off)	0.57	0.16	0.26 to 0.88	< 0.01	0.52	0.19	0.15 to 0.89	< 0.02				
Conventional (dampened)	0.41	0.19	0.04 to 0.78	< 0.05	0.49	0.18	0.14 to 0.84	< 0.02				

*VPT* vibration perception threshold

Inter-rater reliability: Determined by Fleiss’ К (Table 3), the four-site monofilament test (*n* = 44) displayed substantial inter-rater reliability (К = 0.61, 95%CI: 0.45 to 0.77, *p* < 0.01) compared to moderate inter-rater reliability for the 10 site test (К = 0.54, 95%CI: 0.38 to 0.70, *p* = 0.02). There was very little discrepancy between reliability when analysing pairs of raters for the 10-site test, however for the four-site test the reliability was relatively higher for the pooling of the more experienced podiatrists (К: 0.72, 95%CI: 0.53 to 0.91, *p* < 0.01) compared to the pooling of the two Podiatrists with less experience (К: 0.55, 95%CI: 0.31 to 0.78, *p* < 0.01), Table 3.

**Table 3:** Inter-rater reliabilty of neurological tests reported as Cohen’s or Fleiss’ *K* and SE with 95%CI and p-values

Inter-rater reliability between pairs of raters (n = 50)												
	Rater 1 & 2				Rater 1 & 3				Rater 2 & 3			
Test	K	SE	95% CI	P	K	SE	95% CI	P	K	SE	95% CI	P
VPT	0.78	0.9	0.59 to 0.96	< 0.01	0.59	0.13	0.33 to 0.84	< 0.01	0.48	0.14	0.21 to 0.75	< 0.01
Monofilament [4]	0.55	0.12	0.31 to 0.78	< 0.01	0.57	0.12	0.33 to 0.80	< 0.01	0.72	0.10	0.53 to 0.91	< 0.01
Monofilament [10]	0.57	0.14	0.30 to0.84	< 0.01	0.50	0.14	0.18 to 0.82	< 0.01	0.57	0.16	0.17 to 0.83	< 0.01
Inter-rater reliability between 3 raters (n = 50)												
Test	К	SE	95% CI	P								
VPT	0.61	0.08	0.45 to 0.77	< 0.01								
Monofilament [4]	0.61	0.08	0.45 to 0.77	0.01								
Monofilament [10]	0.54	0.08	0.38 to 0.70	0.02								
Inter-rater reliability for tuning fork between 2 raters (n = 24)												
	T1 & T2 (*K*)	SE	95% CI	P								
Conventional (on/off)	0.63	0.17	0.30 to 0.96	< 0.01								
Conventional (dampened)	0.66	0.15	0.37 to 0.95	< 0.01								
Graduated	0.68	0.14	0.41 to 0.95	< 0.01								

*VPT* vibration perception threshold

### Neurothesiometer

Intra-rater reliability: The neurothesiometer (*n* = 50) demonstrated a range of intra-rater reliability from moderate (К = 0.52, 95%CI: 0.21 to 0.82, *p* = 0.01) to substantial (К = 0.78, 95%CI: 0.58–0.98, *p* = 0.02), Table 2.

Inter-rater reliability: Determined by Fleiss’ К, the neurothesiometer (*n* = 44) demonstrated substantial inter-rater reliability (К: 0.61, 95%CI: 0.45 to 0.77, *p* < 0.01). The most experienced pair of raters in this instance produced a substantially lower reliability (К: 0.48, 95%CI: 0.21 to 0.75, *p* < 0.01) compared with the least experienced pair (К: 0.78, 95%CI: 0.59 to 0.96, *p* < 0.01), Table 3.

### Tuning fork

Intra-rater reliability: The conventional and graduated methods outperformed the dampened method for both the testers R4 and R5 (*n* = 24), though all methods demonstrated moderate intra-rater reliability (К: 0.41 to 0.57), Table 2.

Inter-rater reliability: The graduated tuning fork (k: 0.68, 95%CI: 0.41–0.95, *p* < 0.01) demonstrated slightly higher inter-rater reliability (n = 24) than the dampened method (К: 0.66, 95% CI: 0.37–0.95, *p* < 0.01) and conventional method (К: 0.63, 95% CI: 0.30–0.96, *p* < 0.01), though all demonstrated substantial reliability, Table 3.

## Discussion

The results from our study indicate that monofilament, neurothesiometer and the tuning fork are acceptably reliable methods of testing protective sensation and vibration perception respectively, with some variability demonstrated between inter- and intra-tester reliability as well as with level of clinical experience. Use of a graduated tuning fork or the on/off method using a conventional, non-graduated tuning fork, demonstrated higher reliability than the dampened method and are therefore more appropriate for clinical use. Overall, greater clinician experience resulted in marginally increased reliability of the graduated and conventional (on/off) tuning fork method and substantially increased reliability of the neurothesiometer. Monofilament tests overall, appear to be reliable with clinical experience possibly increasing the reliability of the four-site test. Despite the acceptable levels of reliability demonstrated by these tests, caution must be used in relying on any one test in isolation. Moderate reliability for example still indicates a marked margin of error in test interpretation and it is axiomatic that clinical tests that have the potential to change clinical practice and drive treatment strategies should strive for higher reliability. When considering using these tests for diagnosis and monitoring of DPN we support the current recommendations of using more than one test (e.g. monofilament and tuning fork) as part of a larger screening examination. In addition, we suggest that testing should be performed regularly and repetitively. Of note, our results relate specifically to the reliability of the tests used, i.e. that the results can be replicated, not that they reflect a correct diagnosis of DPN. While use of tests with high reliability is essential for effective clinical management, so too is the need for the tests to be able to diagnose the target condition. It has been stated that two-test combinations have > 87% sensitivity in detecting DPN [36], though further work to determine the combination test with highest reliability that is most diagnostically accurate for identifying presence of DPN is required.

Previous investigation into the 10 g monofilament has shown mixed reliability. A nine-site monofilament test has been shown to have excellent intra- and inter-reliability [20]. Meijer et al., described moderate to good intra-rater and good inter-rater reliability, respectively, for a two-site test [21] while a three-site test has demonstrated fair to moderate inter- and intra-reliability [37]. Lastly, level of agreement between the four- and 10-site test in 1915 people with diabetes was recently shown to be high (К: 0.87) [26] indicating that these tests may be similarly reliable. Our study supports the relatively high inter-rater reliability of the four- And 10-site 10 g monofilament tests previously reported. The inter-rater reliability of four- and 10-site tests from this present study demonstrated similar levels of reliability overall, although experience improved reliability for the four-site test. The excellent intra-rater reliability previously described in the nine-site monofilament test [20] was not replicated in the four or 10 site tests used in our study. The large range of intra-rater reliability of the monofilament (fair to substantial) was not associated with greater clinical experience. As these tests rely on subjective responses from a patient, it is possible that these tests will demonstrate variability regardless of the level of experience of the clinician.

The reliability of a variety of methods of assessing vibration perception was determined in this study including an on/off and a dampening method of a conventional, non-graduated tuning fork, a graduated tuning fork and the neurothesiometer. Of these, the neurothesiometer (*n* = 50) demonstrated the highest intra-rater reliability and the graduated tuning fork (*n* = 24) the highest inter-rater reliability. The reliability demonstrated may have been affected by the comparatively low participant numbers in the tuning fork cohort. Overall, the inter-rater reliability of vibration tests was substantial. Our findings regarding the neurothesiometer are supported by two smaller studies investigating the neurothesiometer [22], biothesiometer and Maxivibrometer [25], respectively. In our study, intra-tester reliability of the neurothesiometer was affected by experience, with the new graduate demonstrating substantially lower reliability (К = 0.52) than the more experienced clinicians (К = 0.72–0.78).

While all tuning fork methods demonstrated substantial inter-rater reliability, the intra-rater reliability was moderate for all methods, and bordering on fair for the dampened method. Previous investigation by Meijer et al., reported substantial intra-rater reliability of the conventional (on/off) method (K = 0.69) at the hallux interphalangeal joint [21]. Perkins et al., noted acceptable reliability of the conventional (on/off) method at the hallux dorsum, without reporting a Kappa statistic [23]. Our findings of moderate intra-tester reliability of the graduated tuning fork are somewhat supported by Thivolet et al., who simply stated statistical significance between test and retest at *p* < 0.01 [24]. A slightly smaller study previously reported low, non-significant inter-rater reliability of the graduated tuning fork [22], which contradicts our findings of substantial reliability. However, the site application and methodology was too dissimilar to our present study to draw any meaningful comparisons. Lastly, the graduated and on/off conventional methods were only marginally affected by experience. We therefore suggest using the graduated tuning fork or conventional on/off method of vibration perception as opposed to the dampened method.

### Limitations

Whilst adding to the paucity of research investigating intra- and inter-rater reliability of vibration perception and monofilament testing in people with diabetes, findings of this study need to be considered in light of several limitations. Though 50 participants attended for test and retest of monofilament and neurothesiometer, only 24 were involved in tuning fork testing. As *n* ≥ 30 is required to satisfy the assumption of normal distribution [38], larger sample studies are warranted. Our study is generalisable to people with type 2 diabetes only, however a strength of this study is that it included people with diagnosed DPN making it generalizable to people requiring testing and ongoing monitoring. In addition, more extensive clinician training and clearer instruction to participants may improve reliability. The findings of this study are also limited to peripheral neurological testing with neurothesiometer, tuning forks and 10 g monofilament. Other neurological tests such as pain perception, proprioception, ankle reflexes, temperature perception, light touch perception and two-point discrimination were not investigated but may be reliable and of clinical value.

## Conclusion

Neurological screening is routinely performed by health professionals on patients with diabetes as they are at risk of developing DPN. Our research suggests that the neurothesiometer, four-site and 10-site 10 g monofilament are all acceptably reliable in screening for DPN. If using a tuning fork to test vibration perception then consider quantification using a graduated tuning fork or the conventional (on/off) method in place of the dampened method. The results of this study also indicate there is a need for regular and repetitive testing and that a combination of tests should be used for screening and monitoring of DPN for Podiatrists regardless of experience level. The reliability of alternate neurological screening methods warrants investigation.

## Data Availability

De-identified data is held securely with the senior author.

## Abbreviations

DPN: Diabetic peripheral neuropathy
VPT: Vibration perception threshold
